# RNase P Ribozymes Inhibit the Replication of Human Cytomegalovirus by Targeting Essential Viral Capsid Proteins

**DOI:** 10.3390/v7072775

**Published:** 2015-06-24

**Authors:** Zhu Yang, Michael Reeves, Jun Ye, Phong Trang, Li Zhu, Jingxue Sheng, Yu Wang, Ke Zen, Jianguo Wu, Fenyong Liu

**Affiliations:** 1 Institute of Virology, School of Life Sciences, Nanjing University, Nanjing 210093, China; E-Mails: nooney1986@163.com (Z.Y.); kzen@nju.edu.cn (K.Z.); 2 Taizhou Institute of Virology, Taizhou 225300, China; E-Mail: sin_angel@foxmail.com; 3 Jiangsu Affynigen Biotechnologies, Inc., Taizhou 225300, China; 4 School of Public Health, University of California, Berkeley, CA 94720, USA; E-Mails: mreeves@berkeley.edu (M.R.); phong.trang@berkeley.edu (P.T.); 5 The People’s Hospital of Taizhou, Taizhou 225300, China; E-Mails: junye1965@hotmail.com (J.Y.); lizhu1975@hotmail.com (L.Z.); 6 Program in Comparative Biochemistry, University of California, Berkeley, CA 94720, USA; E-Mail: jxsheng@berkeley.edu; 7 State Key Laboratory of Virology and College of Life Sciences, Wuhan University, Wuhan 430072, China; E-Mail: jwu@whu.edu.cn

**Keywords:** gene therapy, ribozyme, RNase P, cytomegalovirus, gene targeting

## Abstract

An engineered RNase P-based ribozyme variant, which was generated using the *in vitro* selection procedure, was used to target the overlapping mRNA region of two proteins essential for human cytomegalovirus (HCMV) replication: capsid assembly protein (AP) and protease (PR). *In vitro* studies showed that the generated variant, V718-A, cleaved the target AP mRNA sequence efficiently and its activity was about 60-fold higher than that of wild type ribozyme M1-A. Furthermore, we observed a reduction of 98%–99% in AP/PR expression and an inhibition of 50,000 fold in viral growth in cells with V718-A, while a 75% reduction in AP/PR expression and a 500-fold inhibition in viral growth was found in cells with M1-A. Examination of the antiviral effects of the generated ribozyme on the HCMV replication cycle suggested that viral DNA encapsidation was inhibited and as a consequence, viral capsid assembly was blocked when the expression of AP and PR was inhibited by the ribozyme. Thus, our study indicates that the generated ribozyme variant is highly effective in inhibiting HCMV gene expression and blocking viral replication, and suggests that engineered RNase P ribozyme can be potentially developed as a promising gene-targeting agent for anti-HCMV therapy.

## 1. Introduction

Human cytomegalovirus (HCMV) belongs to the herpesvirus family and is a significant pathogen causing severe illness in newborns and immunocompromised populations, such as HIV-infected patients [1]. HCMV is also one of the most common viral pathogens causing birth defects including deafness and mental retardation [2]. However, no vaccine is currently available for preventing HCMV infection and few effective drugs are currently available for anti-HCMV therapy. It is important for researchers to develop novel therapeutic approaches to treat and prevent infections by HCMV.

**Figure 1:**
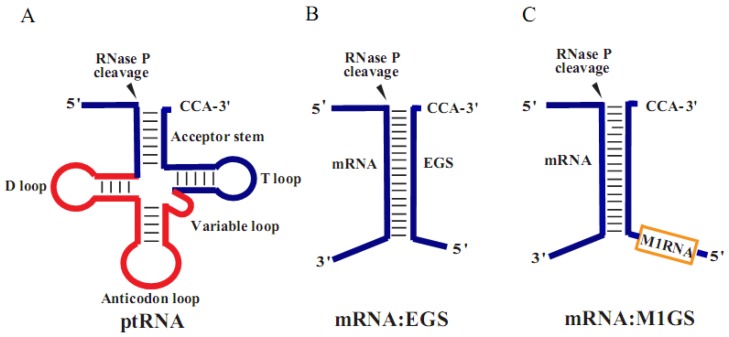
Substrates for RNase P and M1 ribozyme. (**A**) pre-tRNA (ptRNA); (**B**) complex of EGS and target mRNA; (**C**) M1GS RNA binding to its mRNA substrate. Arrowhead indicates the cleavage sites.

RNase P is a ribonucleoprotein complex involving in tRNA processing in cells [3]. In previous studies, researchers have shown that RNase P of *Escherichia coli* contains a catalytically active RNA (M1 RNA) which hydrolyzes different substrates by recognizing tertiary structure (e.g., a stem structure resembling the acceptor stem and T stem regions of a tRNA) rather than primary sequence (Figure 1) [4]. Thus, any mRNA substrate can be potentially cleaved by a custom-designed RNase P-based ribozyme, M1GS, which is generated by covalently linking an external guide sequence (designated as EGS) to the 3′ terminus of M1 RNA (Figure 1) [5,6,7,8].

Gene silencing technologies that target specific RNA sequences of choice, such as antisense oligonucleotide, RNAi, aptamer, microRNA, and ribozyme, represent promising therapeutic strategies [9,10,11,12]. Compared to RNAi and some other gene-targeting approaches, ribozymes have several unique advantages. Unlike the RNAi approach which induces several cellular factors (Exportin V, Drosha, or Dicer) and may affect normal cellular functions [11,13,14], RNase P ribozymes, considered exogenous agents, can be expressed in a wide range of living organisms and can be induced to cleave targeted RNAs [15,16]. Moreover, the catalytic activity and specificity of ribozymes can be easily improved by *in vitro* studies [17,18]. Therefore, ribozyme-based approaches can be developed as powerful tools for both basic research and clinical applications.

Improving RNase P ribozyme catalytic efficiency is one of the most important steps to develop ribozyme-based technology for practical uses. In previous studies, our group has constructed ribozyme variants which are more active in targeting by using an *in vitro* selection procedure [19,20]. In this report, we designed and generated a ribozyme variant, V718-A, to target the overlapping region of HCMV protease (PR) and capsid assembly protein (AP) mRNAs. Both AP and PR, which are encoded by viral UL80.5 and UL80 open reading frames (ORFs) respectively, may be considered ideal antiviral targets since they are highly conserved and are necessary for capsid assembly and viral growth [1,21,22]. We also studied the activity of the generated ribozymes *in vitro* and their efficacy in reducing the expression levels of target genes and viral replication in cultured cells. Results showed that the generated ribozyme variant (V718-A) was more active than wild type ribozyme (M1-A) in inhibiting AP/PR expression and blocking HCMV growth.

## 2. Materials and Methods

### 2.1. Viruses, Cells and Antibodies

HCMV (strain AD169) was propagated in human glioblastoma U251 cells and human foreskin fibroblasts (HFF) which were maintained in DMEM with 10% (*v/v*) FBS [17,20,23,24]. The monoclonal antibodies UL44 (ICP36) and UL83 were purchased from Virusys (Taneytown, MD, USA). The anti-rabbit polyclonal antibodies against HCMV protease (UL80) and capsid assembly protein (UL80.5) were kindly provided by Annette Meyer, Pfizer, Inc. (Ann Arbor, MI, USA), and John Wu of Promab, Inc. (Albany, CA, USA). Other antibodies used in this study were described in previous studies [17,20,23,24].

### 2.2. Mapping of the AP mRNA Accessible Regions in Cells

We carried out an *in vivo* mapping approach to study the accessible regions of AP mRNA following protocols described previously [17,25,26]. First, HCMV-infected cells were treated with dimethyl sulfate (DMS) for 5–10 min, then total RNAs were isolated and used for primer extension assays with radiolabeled oligonucleotides. Finally, primer extension products were separated and analyzed in denaturing gels (8%). The sites modified by DMS represent accessible regions potentially for ribozyme binding.

### 2.3. *In Vitro* Ribozyme Studies

The DNA template of substrate ap11, which contains the 37 nucleotide long AP mRNA sequence, was amplified by PCR using pGEM3zf (+) as a template with forward primer AF25 (5′-GGAATTCTAATACGACTCACTATAG-3′) and reverse primer AP11 (5′-CGGGATCCGTCCGAGGACGACGACGACGCCGCCGCCCTATAGTGAGTCGTATTA-3′) which contains a T7 promoter and the AP coding sequence. Plasmids pFL117, pV718, pV718-C and pC102, which were described in previous studies [19,27], were used as templates to generate ribozymes M1-A, V718-A, V718-C and M1-C, respectively. The forward primer was AF25 while the reverse primer was M1AP11 (5′-CCCGCTCGAGAAAAAATGGTGTCGTCGTCGTCCTCGGATGTGGAATTGTG-3′) with the positions corresponding to the guide sequence underlined. Ribozymes M1-C and V718-C contained the same mutations found in C102 which is a non-functional M1 RNA mutant with point mutations (A_347_C_348_ → C_347_U_348_, C_353_C_354_C_355_G_356_ → G_353_G_354_A_355_U_356_). A T7 *in vitro* transcription kit (Promega) was used for synthesizing RNA substrate ap11 and ribozyme RNAs [28]. Kinetic analyses and gel-shift binding assays were carried out following experimental procedures as described [19,20,29].

### 2.4. Construction of Ribozyme-Expressing Cell Lines

Cell lines expressing ribozymes were constructed as described previously [20,30,31]. Briefly, the M1GS coding sequences were subcloned into retroviral vector LXSN, then the constructed LXSN-M1GS vectors were transfected into PA317 cells. Culture supernatants were harvested at 48 h post-transfection, and used to infect U251 cells. At 48–72 h post-infection, cells were grown in culture medium containing neomycin (600 µg/mL), and neomycin-resistant cells were selected and cloned [20,30,31].

### 2.5. Studies of Viral Gene Expression and Growth in M1GS-Expressing Cells

M1GS-expressing cells were either mock-treated or infected with HCMV at a multiplicity of infection (MOI) of 1. Total RNA and protein samples were prepared at different time points post-infection and studied by Northern and Western blot analyses [31,32,33]. To detect IE2 mRNA and protein expression, samples were harvested at 8 and 24 h post-infection, respectively. To detect the expression of US2 and AP/PR mRNAs, samples were harvested at 48 h post-infection [31,32,33]. To detect the protein expression of UL44, UL83, AP, and PR, samples were harvested at 72 h post-infection. In control experiments, to detection the 5kb RNA expression, samples were harvested at 8 and 48 h post-infection. To detection the actin protein expression, samples were harvested at 24 and 72 h post-infection [31,32,33]. For Northern blot analyses, the prepared RNA samples (30 μg) were separated in denaturing agarose gels (1%) that contained formaldehyde. The separated RNAs were transferred to membranes and hybridized with the [^32^P]-radiolabeled probes complementary to HCMV sequences. For Western analyses, the prepared protein samples (50 μg) were separated on SDS-PAGE gels (7.5%), then transferred to membranes and reacted with the antibodies against human β-actin and HCMV proteins and stained with the aid of an ECL Western blot detection kit (GE Healthcare) [31,32].

To determine the ribozyme-directed reduction of HCMV growth, U251 cells (*n* = 1 × 10^5^) expressing M1GSs were infected with HCMV (MOI = 1). The medium and cells were collected at1-day intervals throughout the 7 days post-infection to prepare viral stocks, and then plaque assays were carried out to study the viral titers of the prepared stocks [31,32].

### 2.6. Determination of the HCMV DNA Level

Total (no DNase I-treated) and encapsidated (DNase I- treated) DNA samples were prepared from cells at 96 h post-infection following the protocols described previously [34,35]. Using the PCR amplified products of actin sequence (610 bp) as the control, the amount of viral DNA was determined by PCR amplification of the HCMV IE1 sequence (481 bp) in which primers CMV3 (5′-CCAAGCGGCCTCTGATAACCAAGCC-3′) and CMV4 (5′-CAGCACCATCCTCCTCTTCCTCTGG-3′) and experimental procedures have previously been described in detail [24,35]. PCR reactions were performed with α-[^32^P]-dCTP, and then the radiolabeled PCR products were separated and analyzed in polyacrylamide gels [24,35].

### 2.7. Statistical Analysis

Experiments were carried out in triplicate, and repeated three times. Statistical analyses of the data were performed using the analysis of variance (ANOVA). Values of *p* ≤ 0.05 were considered statistically significant.

## 3. Results

### 3.1. Ribozyme-Mediated Cleavage of the AP mRNA Sequence in Vitro

Most intracellular mRNAs are folded in complex structures in cells, so it is critical to choose an accessible region for ribozyme binding to achieve highly efficient cleavage [8,35]. In this study, an *in vivo* mapping approach was used to study the accessible AP mRNA regions in HCMV-infected cells. The position 548 nucleotides downstream of the AP translation start site [36,37] appeared to be extremely permissive for DMS modifying (data not shown). Hence, this region was chosen as the target site for the guide sequence. Moreover, its flanking sequence contains elements that are known to be important for efficient M1GS-mediated cleavage [29,38].

In previous studies, we generated ribozyme variants that are more efficient than wild type ribozyme in targeting the HSV-1 thymidine kinase (TK) mRNA sequence [19,39]. However, whether these generated variants are also highly active in cleaving HCMV mRNA to inhibit viral growth has not been extensively investigated. In this report, we chose to characterize the activity of variant 718 (designated as V718), which has been generated by *in vitro* selection procedure and has not been studied before [19]. This variant has two point mutations (*i.e.*, A_81_ → C_81_ and G_194_ → A_194_) and is efficient in cleaving AP mRNA sequences *in vitro* (Table 1).

**Table 1:** Kinetic parameters [(k_cat_/K_m_)^s^ and K_d_] of different ribozymes-mediated cleavage with substrate ap11.

Enzyme	(k_cat_/K_m_)^s^ (µM^−1^·min^−1^)	K_d_ (nM)
M1-A	0.22 ± 0.05	0.31 ± 0.05
V718-A	13.5 ± 2.5	0.35 ± 0.06
M1-C	<5 × 10**^−^**^6^	0.32 ± 0.06
V718-C	<5 × 10**^−^**^6^	0.34 ± 0.05
M1-TK	<5 × 10**^−^**^6^	ND

Values are means of triplicate determinations. *p* < 0.02*.* “ND”, not detected.

Active ribozymes V718-A and M1-A were constructed from V718 and M1 RNA, respectively. Control ribozymes M1-C and V718-C, which were generated from an inactive ribozyme mutant C102 in a similar way, were expected to be inactive due to mutations in the catalytic domain [27,33]. These four ribozymes all contained the same guide sequence complementary to the target AP mRNA sequence. We carried out *in vitro* kinetic analyses to study the catalytic activity (k_cat_/K_m_)^s^ of different ribozymes in cleaving substrate ap11, which contained the 37 nucleotide long AP mRNA sequence. Our results indicated that V718-A activity was about 60-fold higher than that of M1-A in targeting substrate ap11 (*p* < 0.02). In contrast, V718-C and M1-C were 10^4^-fold less effective than M1-A in targeting substrate ap11 (*p* < 0.02) (Table 1). To study whether the differential cleavage efficiencies of these ribozymes were possibly caused by different binding affinities, the binding affinities of ribozymes to the substrate ap11, measured as the dissociation constant (K_d_), were studied by gel-shift assays. The binding affinities of V718-C and M1-C were similar to those of V718-A and M1-A (Table 1). Therefore, V718-C and M1-C could be used as antisense effect controls since V718-C and M1-C are inactive ribozymes but have similar binding affinity to ap11 as V718-A and M1-A.

### 3.2. Ribozyme Expression in the Cultured Cells

The DNA sequences coding for V718-A, M1-A, V718-C, and M1-C were subcloned into retroviral vector LXSN which was used to express M1GS RNAs successfully in previous studies [8,33,40]. The constructed LXSN-M1GS vector DNAs were transfected into PA317 cells to produce vector particles. These vectors were then harvested and used to infect human U251 cells, and cell lines expressing M1GSs were cloned. Another cell line expressing M1-TK ribozyme which was generated to cleave the HSV-1 TK mRNA was also generated, and used as the control for the ribozyme with mismatched guide sequences [19,20,39]. As shown in our results, ribozyme M1-TK had no catalytic activity with substrate ap11 *in vitro* (data not shown, Table 1).

**Figure 2:**
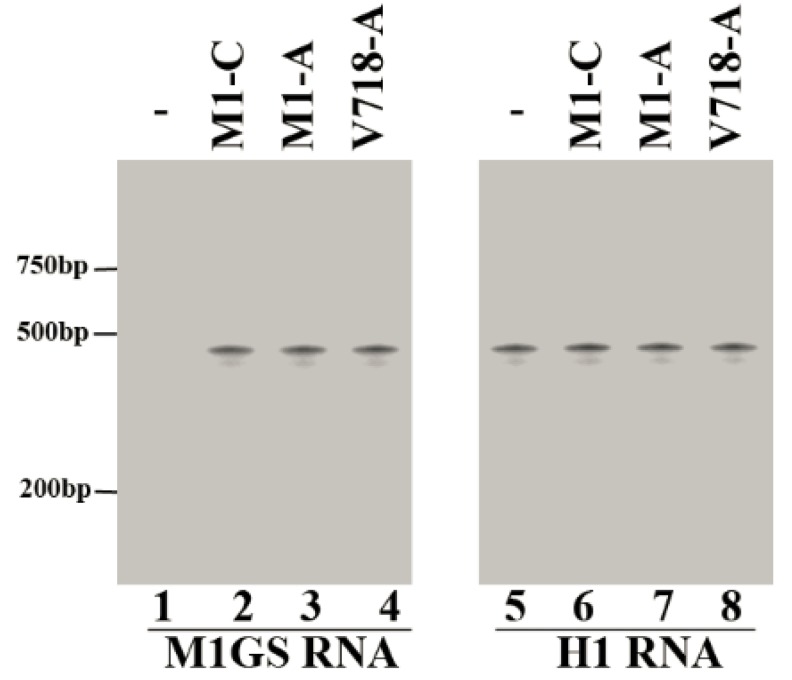
Expression levels of ribozymes in cultured cells. Northern blot analyses were performed using RNA samples (30 µg) prepared from parental U251 cells (-, lanes 1 and 5) and cells expressing M1-C (lanes 2 and 6), M1-A (lanes 3 and 7), and V718-A (lanes 4 and 8). Human H1 RNA control (lanes 5–8) (the RNA subunit of human RNase P and a nuclear RNA) [31,41].

The expression levels of M1GS RNA in the cells were studied by Northern blot analyses (Figure 2, lanes 1–4), using human H1 RNA as the internal control (Figure 2, lanes 5–8). Only cell clones expressing ribozymes at similar levels were chosen and used for subsequent experiments.

### 3.3. Ribozyme-Mediated Reduction of HCMV AP and PR Expression

**Figure 3:**
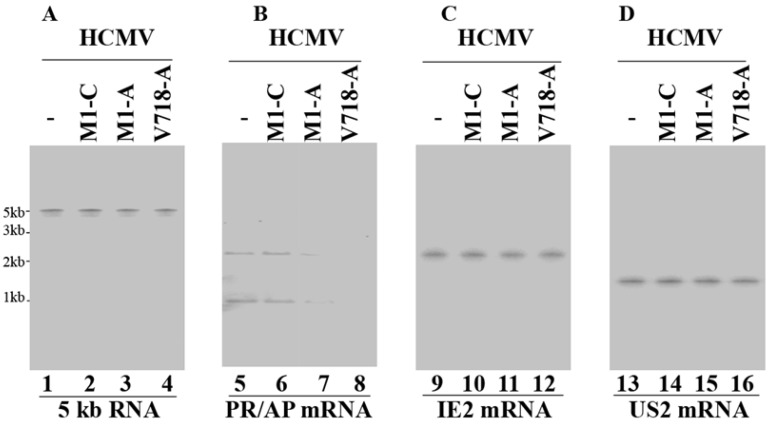
Northern blot analyses of the expression levels of HCMV mRNAs. RNA samples (30 µg) were isolated from HCMV-infected parental U251 cells (-, lanes 1, 5, 9, and 13) and cell lines expressing M1-C (lanes 2, 6, 10, and 14), M1-A (lanes 3, 7, 11, and 15), and V718-A (lanes 4, 8, 12, and 16) at 8 h (**C**) and 48 h post-infection ((**A**), (**B**), and (**D**)). The hybridized probes were used to detect the HCMV 5 kb RNA (lanes 1–4), AP/PR mRNA (lanes 5–8), IE2 mRNA (lanes 9–12), and US2 mRNA (lanes 13–16).

Northern blot analyses were performed to study AP and PR mRNAs expression levels, using HCMV immediate-early 5 kb RNA (5 kb RNA) as the internal and loading control (Figure 3, lanes 1–4). A reduction of 98%–99% and 75%–78% (*p* < 0.04) in AP/PR mRNA expression was observed in the cells expressing V718-A and M1-A, respectively (Figure 3, lanes 7 and 8). In contrast, cells expressing control ribozyme M1-C and V718-C showed no significant reduction (<10%) (Figure 3, lanes 6, data not shown) (Table 2). All of the results suggested that inhibition of AP/PR mRNA expression in the cells with V718-A and M1-A was due to ribozyme-directed cleavage of AP/PR mRNAs.

**Figure 4:**
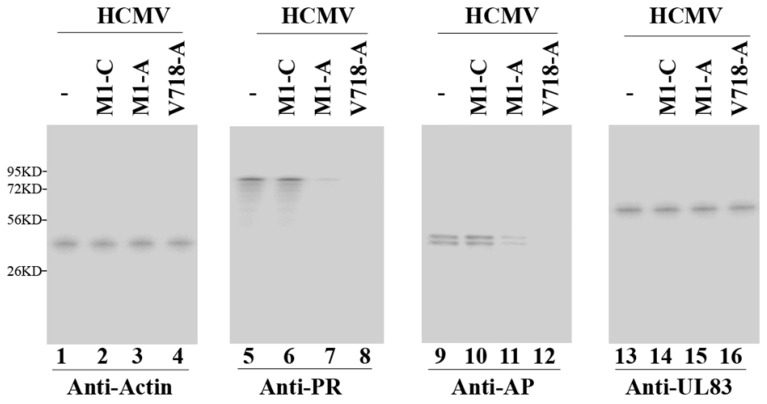
Western blot analyses of the expression levels of HCMV proteins. Protein samples (50 µg) were prepared at 72 h post-infection and then separated in SDS-PAGE gels, transferred to membranes and reacted with antibodies. The anti-AP antibody detected both full-length AP and its processed form that is cleaved during HCMV capsid assembly [1] (lanes 9–12). Actin control (lanes 1–4).

Western blot analyses were performed to study the expression levels of AP and PR proteins, using actin as the internal and loading control (Figure 4, lanes 1–4). A reduction of about 98%–99% and 72%–75% (*p* < 0.04) in the PR and AP protein expression levels was observed in cells with V718-A and M1-A (Figure 4, lanes 7, 8, 11, and 12) (Table 2). In comparison, little reduction (<10%) was observed in cells expressing M1-C, V718-C, or M1-TK (Figure 4, lanes 6 and 10, data not shown) (Table 2). Thus, the decrease in the AP and PR protein expression correlated with the reduction of the AP and PR mRNA expression.

**Table 2:** Inhibition Levels of HCMV mRNA and protein expression in the cells with M1GS, as compared with parental U251 cells.

	Viral Gene Class	Ribozymes					
		U251	M1-C	V718-C	M1-A	V718-A	M1-TK
IE2 mRNA	α	0%	0%	0%	0%	0%	0%
US2 mRNA	β	0%	0%	0%	0%	0%	0%
AP mRNA	γ	0%	3%	6%	78% ± 8%	99% ± 7%	0%
PR mRNA	γ	0%	3%	7%	75% ± 7%	98% ± 7%	0%
IE2 protein	α	0%	0%	0%	0%	0%	0%
UL44 protein	β, γ	0%	0%	0%	0%	0%	0%
UL83 protein	γ	0%	0%	0%	0%	0%	0%
AP protein	γ	0%	3%	6%	75% ± 7%	99% ± 8%	0%
PR protein	γ	0%	3%	6%	72% ± 7%	98% ± 8%	0%

Experiments were carried out in triplicate, and repeated three times. Values are means from these experiments and standard deviations less than 5% were not recorded. Samples were harvested at different time points post-infection as specified in Materials and Methods.

### 3.4. Inhibition of HCMV Replication Mediated by the Ribozymes

**Figure 5:**
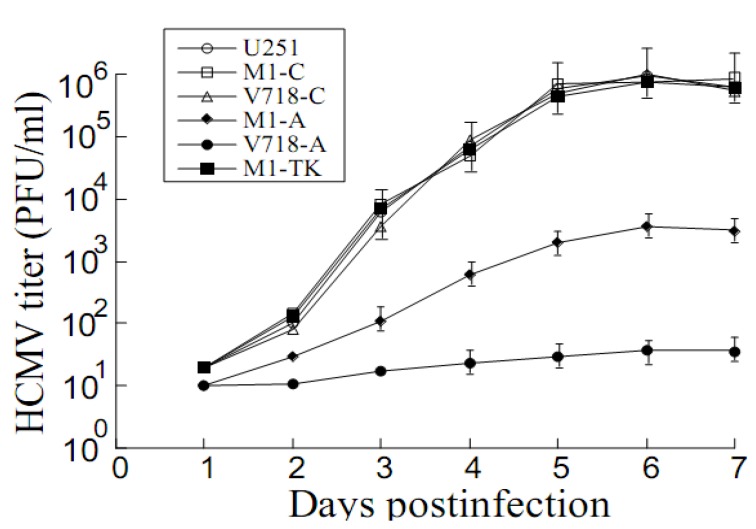
HCMV growth in U251 cells and M1GS-expressing cell lines. Experiments were carried out in triplicate, and repeated three times. Values are means of these experiments and the standard deviation is indicated by the error bars.

Plaque assays were performed to examine M1GS-mediated inhibition of HCMV replication. A 50,000 and 500-fold decrease in viral titers was found in cells with V718-A and M1-A, respectively (Figure 5). Little reduction was observed in cell lines with control ribozymes V718-C, M1-C, or M1-TK (Figure 5). The results suggested that the generated ribozymes inhibited viral PR and AP expression and blocked HCMV replication.

### 3.5. The Antiviral Mechanism of Ribozyme-Mediated Cleavage of AP and PR mRNAs

HCMV assembly protein (AP) and protease (PR) play essential roles in DNA encapsidation during capsid assembly and viral growth [1,21,22]. Therefore, we carried out two sets of experiments to study the relationships between the ribozyme-mediated cleavage of the target genes and HCMV lytic replication cycle.

First, we determined whether the generated ribozymes have effect on the expression levels of other viral genes (except AP and PR) in the cells. Northern blot analyses were performed to examine the expression of other viral genes, such as IE2 (an immediate-early (IE) or α gene) and US2 (an early or β gene) mRNA in cells with M1GS (Figure 3, lanes 9–12 and 13–16). Furthermore, Western blot analyses were also carried out to study the expression levels of viral proteins UL44 (an early/late or βγ gene) and UL83 (a late or γ gene) (Figure 4, lanes 13–16, data not shown) (Table 2). No difference was found in the expression levels of these genes in cells with different ribozymes (V718-A, M1-A, V718-C, and M1-C) (Table 2). Thus, V718-A and M1-A specifically inhibited AP and PR expression, and had no effect on overall viral gene expression.

**Figure 6:**
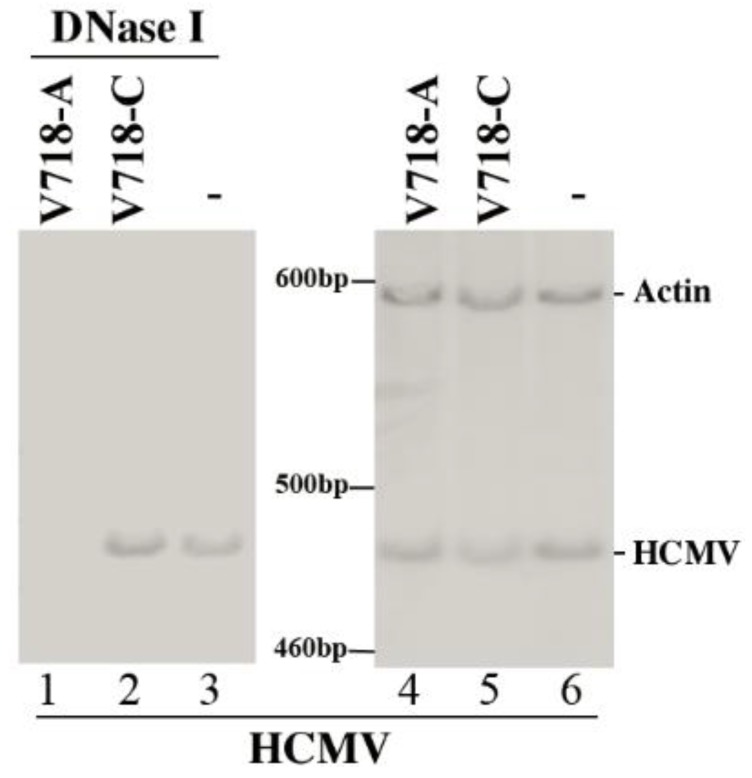
The levels of HCMV encapsidated DNA and total intracellular DNA. DNase I-treated DNA samples (lanes 1–3) or total DNA (lanes 4–6) were prepared from cells without ribozyme (-, lanes 3 and 6) or with ribozyme V718-A (lanes 1 and 4) and V718-C (lanes 2 and 5) at 96 h post-infection. Semi-quantitative PCR was performed to determine the levels of viral IE1 sequence and human actin DNA sequence was used as the internal control.

Second, we examined whether the generated ribozymes have effect on HCMV DNA replication or capsid maturation. DNA was prepared from samples treated either with or without DNase I since uncapsidated DNA would be susceptible to DNase I treatment, and then the amounts of viral DNA were determined. Results showed that the levels of total intracellular HCMV DNA (no DNase I-treatment) were similar in the cells with different ribozymes (Figure 6, lanes 4–6). In contrast, the levels of “encapsidated” DNA (DNase I-treated) were greatly decreased in the cells with V718-A (Figure 6, lane 1). The results indicate that M1GS-mediated reduction of AP and PR expression has no effect on HCMV genome replication but inhibits DNA encapsidation and capsid formation.

## 4. Discussion

In recent years, gene silencing technologies, such as antisense oligonucleotides, RNAi, aptamers, and microRNAs, have been widely used for research studies [9,10,11]. However, each technology has disadvantages such as specificity, activity, stability and cellular toxicity [13,18]. Compared to other approaches, RNase P ribozyme may represent a promising and attractive approach with high activity and specificity [3,41,42]. In this study, M1GS RNAs were generated to target the overlapping region of HCMV PR (UL80) and AP (UL80.5) mRNAs. Further studies showed that the expression levels of PR (UL80) and AP (UL80.5) were greatly decreased (98%–99%), and viral growth was also largely reduced (50,000-fold) in the cells expressing V718-A. In comparison, little reduction (<10%) was found in AP and PR expression and viral replication in the cells with control ribozymes (*i.e.*, V718-C, M1-C, or M1-TK). The results indicate that V718-A is highly active in reducing HCMV gene expression and blocking viral growth.

Evidences presented in this study suggest that the antiviral effects of ribozyme are probably resulted from ribozyme targeting of AP/PR mRNAs. First, the generated ribozymes (*i.e.*, V718-A, M1-A) only inhibited the expression of AP and PR; no decrease was found in the expression levels of viral α, β, or γ genes examined (e.g., IE2, US2, UL44 and UL83) (Figure 3 and Figure 4, Table 2, data not shown). Second, viral genomic DNA replication was not affected by ribozyme expression (Figure 6). Third, blocking of capsid maturation and viral growth appeared to be induced by ribozyme-mediated cleavage of AP and PR mRNAs, since encapsidated viral DNA levels as well as AP/PR expression levels were found to be reduced in the cells with V718-A and M1-A but not in the cells with V718-C, M1-C, or M1-TK (Figure 3, Figure 4, Figure 5 and Figure 6 data not shown). All together, these resultssuggest that AP and PR play necessary roles in viral capsid maturation and have no effect on HCMV gene expression or genome replication [1,22,43].

Further studies on improving the cleavage efficacy of M1GS RNAs are important to develop ribozyme-based approaches [23,39,42]. However, no guidelines are currently available about how to generate a highly active M1GS RNA [23,39,40]. In this study, functional ribozymes (e.g., V718-A) were designed to target an accessible region of the overlapping region of AP and PR mRNAs. With this design, we hypothesized that the M1GS cleavage efficacy in cells is determined by its catalytic efficiency [(k_cat_/K_m_)^s^] *in vitro*. If this is the case, improving the catalytic activity of ribozymes [(k_cat_/K_m_)^s^] may lead to more active ribozyme-directed cleavage in cells [23,39]. As shown in our studies, an M1GS variant (*i.e.*, V718-A) was about 60 times more active [(k_cat_/K_m_)^s^] in targeting AP and PR mRNAs *in vitro* than the wild type ribozyme (*i.e.*, M1-A). V718-A was also more effective than M1-A in reducing AP and PR expression in cultured cells (e.g., 98%–99% *vs*. 72%–75%). In comparison, little reduction (<10%) was observed in the expression levels of AP and PR and in viral replication in cells with V718-C, M1-C, or M1-TK. All of these results support our hypothesis that improving the *in vitro* catalytic efficiency [(k_cat_/K_m_)^s^] may lead to increased cleavage activity of ribozymes in cells. Therefore, our work provides insights into developing guidelines for the generation of highly active ribozymes.

Our previous studies have shown that ribozymes derived from M1 RNA variants could be constructed to target the mRNAs coding for various cytomegalovirus (CMV) essential proteins including immediate early protein 1 (IE1) and 2 (IE2), AP, and PR [20,28,29,33]. These ribozymes efficiently cleaved the target mRNAs *in vitro* and effectively blocked HCMV gene expression and replication in cultured cells. In our current study, we reported the anti-HCMV activity of ribozymes derived from a M1 RNA variant, V718, which has been generated by an *in vitro* selection procedure and has not been characterized before. V718-A appeared to cleave the AP/PR mRNAs more efficiently than M1-A *in vitro* (Table 1). Furthermore, V718-A more effectively inhibited HCMV gene expression and growth than M1-A in cultured cells. The extent of antiviral effects observed in V718-A expressing cells (*i.e.*, the level of inhibition of viral gene expression and growth) is at least 10 times higher than that observed in cells expressing a ribozyme variant targeting HCMV IE2, which was recently reported by our laboratory [33]. Indeed, antiviral effects associated with V718-A are among the best effects observed in our studies using different ribozyme variants that target different CMV targets including AP and PR [20,28,29,33]. The ribozyme variant V718 has two point mutations: A_81_ → C_81_ and G_194_ → A_194_. How these two mutations increase cleavage activity is not known. Further studies on the mutations found in V718 and other variants will provide insight into the mRNA cleaving mechanism of the ribozymes.

HCMV is a human herpesvirus and causes significant mortality and morbidity in specific human populations [1]. HCMV protease (UL80) and capsid assembly (UL80.5) proteins are necessary for capsid assembly and viral growth, suggesting that these proteins could be used as ideal targets for anti-HCMV development [17,24]. HCMV is known to infect and replicate in many types of cells and tissues *in vivo* [1]. In this report, we showed that V718-A effectively inhibited AP/PR expression and blocked HCMV growth in human astrocytoma U251 cells that were infected with HCMV at a modest multiplicity of infection (MOI). It is important to determine if V718-A effectively inhibits HCMV gene expression and replication in other cells and tissues known to be infected by HCMV *in vivo* and under different MOIs. Additional studies on V718 and other ribozymes should provide insights into developing active ribozymes for anti-HCMV therapeutic applications. 

## 5. Conclusions

HCMV is an opportunistic pathogen that establishes a life-long infection in infected individuals. Developing effective and novel drugs are important for controlling and treating HCMV infection. In this report, we studied the antiviral effect of an engineered ribozyme variant, V718-A, in cultured cells. As shown by our results, V718-A had about 60-fold higher cleavage activity than M1-A *in vitro*. Furthermore, HCMV growth was reduced by 50,000 fold in the cells expressing V718-A. Our study implies that the generated ribozyme variant, V718-A, is highly active in reducing HCMV AP/PR gene expression and blocking viral replication, and suggests that the generated V718-A can be potentially developed as a gene-targeting agent for anti-HCMV therapy.
